# TLR4-Mediated Inflammatory Responses Regulate Exercise-Induced Molecular Adaptations in Mouse Skeletal Muscle

**DOI:** 10.3390/ijms23031877

**Published:** 2022-02-07

**Authors:** Haruna Fujiyoshi, Tatsuro Egawa, Eriko Kurogi, Takumi Yokokawa, Kohei Kido, Tatsuya Hayashi

**Affiliations:** 1 Laboratory of Sports and Exercise Medicine, Graduate School of Human and Environmental Studies, Kyoto University, Kyoto 606-8501, Japan; fujiyoshi.haruna.32z@st.kyoto-u.ac.jp (H.F.); kurogi.eriko.57m@st.kyoto-u.ac.jp (E.K.); tatsuya@kuhp.kyoto-u.ac.jp (T.H.); 2 Laboratory of Health and Exercise Sciences, Graduate School of Human and Environmental Studies, Kyoto University, Kyoto 606-8501, Japan; 3 Division of Food Science and Biotechnology, Graduate School of Agriculture, Kyoto University, Kyoto 606-8502, Japan; yokokawa.takumi.8w@kyoto-u.ac.jp; 4 Faculty of Sports and Health Science, Fukuoka University, Fukuoka 814-0180, Japan; kohei.kido110@gmail.com; 5 Institute for Physical Activity, Fukuoka University, Fukuoka 814-0180, Japan

**Keywords:** endurance exercise, inflammation, mitochondrial biogenesis, PPARβ, HSP72

## Abstract

Endurance exercise induces various adaptations that yield health benefits; however, the underlying molecular mechanism has not been fully elucidated. Given that it has recently been accepted that inflammatory responses are required for a specific muscle adaptation after exercise, this study investigated whether toll-like receptor (TLR) 4, a pattern recognition receptor that induces proinflammatory cytokines, is responsible for exercise-induced adaptations in mouse skeletal muscle. The TLR4 mutant (TLR4m) and intact TLR4 control mice were each divided into 2 groups (sedentary and voluntary wheel running) and were housed for six weeks. Next, we removed the plantaris muscle and evaluated the expression of cytokines and muscle regulators. Exercise increased cytokine expression in the controls, whereas a smaller increase was observed in the TLR4m mice. Mitochondrial markers and mitochondrial biogenesis inducers, including peroxisome proliferator-activated receptor beta and heat shock protein 72, were increased in the exercised controls, whereas this upregulation was attenuated in the TLR4m mice. In contrast, exercise increased the expression of molecules such as peroxisome proliferator-activated receptor-gamma coactivator 1-alpha and glucose transporter 4 in both the controls and TLR4m mice. Our findings indicate that exercise adaptations such as mitochondrial biogenesis are mediated via TLR4, and that TLR4-mediated inflammatory responses could be involved in the mechanism of adaptation.

## 1. Introduction

Endurance exercise induces various types of adaptations and contributes to health, especially in skeletal muscles, which constitute approximately 40% of body weight. A short period of intense exercise increases glucose uptake and energy consumption in skeletal muscle [1,2], and continuous exercise training increases insulin sensitivity [3,4] and mitochondrial biogenesis [5,6,7]. This exercise-induced mitochondrial adaptation leads to higher oxidative capacity, and the energy supply system changes from glycolytic to oxidative [5,8], thereby adapting skeletal muscles to endurance exercise. In today’s sedentary society, exercise-induced skeletal muscle adaptations are important because they confer health benefits; however, the molecular mechanism has not been completely elucidated.

Inflammation is a natural defensive reaction of the immune system caused by various types of stimulation, including tissue injury, invasion by microorganisms, virus infection, chemical exposure, food intake, and exercise. Regarding the inflammatory response to exercise, levels of proinflammatory cytokines, such as tumor necrosis factor alpha (TNF-α) and interleukin (IL)-1β, have been reported to increase after exercise and induce muscle proteolysis and oxidative damage [9,10]. Inflammation has historically been considered a detrimental process associated with muscle damage, pain, and delayed recovery [11], and treatments such as icing and medicine have been commonly used to control inflammation. However, several studies have demonstrated that inflammation is a key process underlying muscular repair and regeneration after exercise [12,13,14]. Accordingly, a recent study has shown that icing of skeletal muscle after injury delayed inflammation, which blunts the efficiency of muscle regeneration [15].

Toll-like receptor (TLR) 4 is a member of the TLR family of pattern recognition receptors that induces proinflammatory cytokine production [16] and is expressed in numerous tissues, including liver tissue, adipose tissue, and skeletal muscle [17]. Various TLR4 ligands have been described, including lipopolysaccharide (LPS) from Gram-negative bacteria [18,19] and saturated free fatty acids [20,21]. TLR4 combined with its ligands activates nuclear factor-κB signaling and produces proinflammatory cytokines, such as TNF-α, IL-1β, and IL-6 [16,22]. Previous studies have suggested that TLR4 mediates an exercise-induced increase in substrate oxidation and mitochondrial enzymatic activity [23] and mediates a signal that links endurance exercise to adaptations by mitogen-activated protein kinase in skeletal muscle [24]. It has therefore been suggested that TLR4-mediated inflammatory responses are critical for skeletal muscle adaptations following exercise. However, it is unclear whether TLR4 mediates an exercise-induced proinflammatory cytokine response and molecular adaptation associated with energy metabolism in skeletal muscle. This study therefore compared the skeletal muscle adaptations of TLR4-inactive (TLR4 mutant [TLR4m]) mice to voluntary wheel running exercise with those of TLR4-active control mice.

## 2. Results

### 2.1. Inflammatory Response to Lipopolysaccharide Was Suppressed in TLR4m Mice

**Figure 1:**
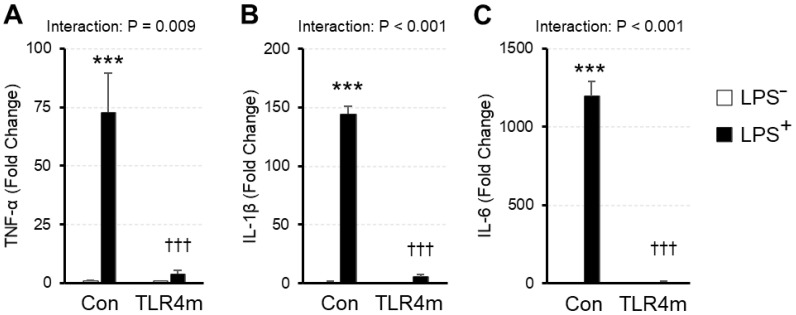
mRNA expression of proinflammatory cytokines in the plantaris with or without lipopolysaccharide (LPS) injection. Two strains of mice, TLR4 mutant (TLR4m) and controls (Con), were divided into groups without LPS injection (LPS−) and LPS (0.4 mg/kg body weight) with LPS injection (LPS+), respectively, and tumor necrosis factor alpha (TNF-α) (**A**), interleukin (IL)-1β (**B**), and IL-6 (**C**) levels were measured. Values are means ± standard error; *n* = 2–3 per group. Fold changes are expressed relative to the level of the control LPS− group. *** *p* < 0.001 vs. LPS− within each strain; ††† *p* < 0.001 vs. controls within each condition.

To confirm whether the skeletal muscle inflammatory response of the TLR4m mice was defective, we preliminarily examined the mRNA expression of proinflammatory cytokines in response to LPS (Figure 1). There was a significant interaction between the LPS injection and strain in TNF-α (*p* = 0.00986) (Figure 1A), IL-1β (*p* < 0.001) (Figure 1B), and IL-6 (*p* < 0.001) (Figure 1C). mRNA expression was remarkably increased after LPS injection in the controls, whereas the increased effects were attenuated in the TLR4m mice.

### 2.2. Body Weight, Plantaris Weight, and Epididymal Fat Weight

There was no statistically significant difference in initial body weight between the strains (*p* = 0.07). Six weeks after the experiment, there was a statistically significant main effect for strain (*p* < 0.001) but not for exercise (*p* = 0.19) on the final body weight; the final body weight of the TLR4m mice was less than that of the controls. Although there was no significant main effect for strain on plantaris weight normalized to body weight (*p* = 0.47) or epididymal fat weight normalized to body weight (*p* = 0.27), there was a significant main effect for exercise on plantaris weight normalized to body weight (*p* = 0.006) and epididymal fat weight normalized to body weight (*p* < 0.001). Exercise training increased the plantaris weight per body weight and decreased epididymal fat weight per body weight (Table 1).

### 2.3. Exercised-Induced Cytokine Expression in Skeletal Muscle Was Attenuated in TLR4m Mice

**Figure 2:**
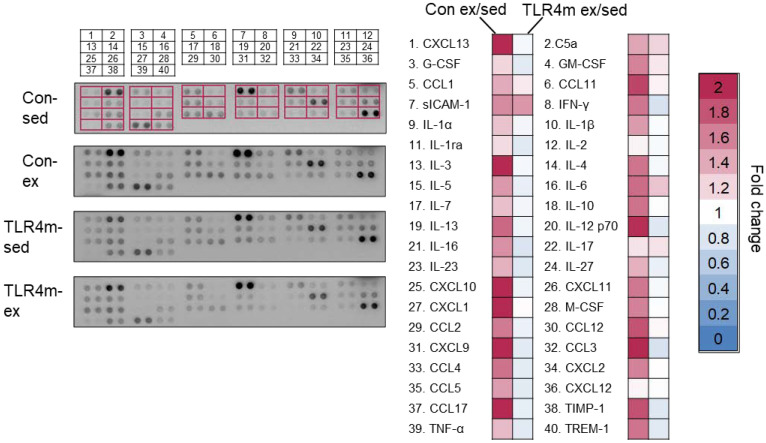
The relative expressions of 40 cytokines in the plantaris. Two strains of mice, TLR4 mutant (TLR4m) and controls (Con), were divided into a voluntary wheel-running group (ex) and a sedentary group (sed), respectively, and housed for six weeks. The arrangement of the 40 cytokines on the membranes is shown in the figure above. The relative values of ex to sed within each strain are represented as a heat map. *n* = 3 per group; Red, increased; Blue, decreased. CXCL, C-X-C motif chemokine ligand; C5a, complement component 5a; G-CSF, granulocyte colony stimulating factor GM-CSF, granulocyte macrophage colony-stimulating factor; CCL, chemokine (C-C motif) ligand; sICAM, soluble intercellular adhesion molecule; INF, interferon; IL, interleukin; M-CSF, macrophage colony stimulating factor; TIMP, tissue inhibitors of metalloproteinase; TNF, tumor necrosis factor; TREM, triggering receptor expressed on myeloid cell.

We investigated the relative expressions of 40 mouse cytokines using the Mouse Cytokine Array Kit to evaluate the inflammatory response to exercise in skeletal muscle. As shown in the heatmap, exercise upregulated the expression of various cytokines in the controls’ plantaris, but little increase was observed in the TLR4m mice (Figure 2).

### 2.4. The Adaptation of Exercise-Induced Mitochondrial Markers in Skeletal Muscle Was Reduced in TLR4m Mice

**Figure 3:**
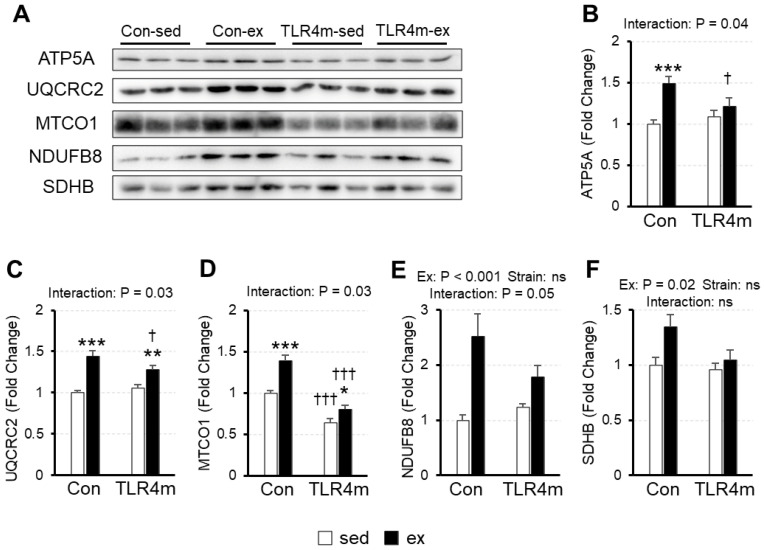
The relative expressions of mitochondrial oxidative phosphorylation proteins in plantaris. ATP synthase subunit alpha (ATP5A) (**B**), cytochrome b-c1 complex subunit 2 (UQCRC2) (**C**), mitochondrial cytochrome c oxidase 1 (MTCO1) (**D**), NADH dehydrogenase [ubiquinone] 1 beta sub-complex subunit 8 (NDUFB8) (**E**), and succinate dehydrogenase [ubiquinone] iron-sulfur subunit (SDHB) (**F**) were measured. Representative images of immunoblots are shown (**A**). Values are means ± standard error; *n* = 6 per group. Fold changes are expressed relative to the level of the Con sed group. *** *p* < 0.001, ** *p* < 0.01, and * *p* < 0.05 vs. sed within each strain; ††† *p* < 0.001 and † *p* < 0.05 vs. controls within each condition.

To examine the exercise-induced mitochondrial adaptations in skeletal muscle, we measured the expression of the mitochondrial oxidative phosphorylation proteins adenosine triphosphate (ATP) synthase subunit alpha (ATP5A); cytochrome b-c1 complex subunit 2 (UQCRC2); mitochondrial cytochrome c oxidase 1 (MTCO1); and NADH dehydrogenase [ubiquinone] 1 beta sub-complex subunit 8 (NDUFB8), as well as succinate dehydrogenase [ubiquinone] iron-sulfur subunit (SDHB) in the plantaris by western blot analysis (Figure 3). There was a significant interaction between exercise and strain on ATP5A (*p* = 0.04) (Figure 3B), UQCRC2 (*p* = 0.03) (Figure 3C), and MTCO1 (*p* = 0.03) (Figure 3D), and a tendency for interaction on NDUFB8 (*p* = 0.051) (Figure 3E); exercise training increased the expression of these proteins in the controls, whereas the increased effects were attenuated in the TLR4m mice. There was a significant main effect of exercise (*p* = 0.02) but not of strain on SDHB (*p* = 0.02) (Figure 3F); exercise increased this protein’s expression. On the other hand, exercise training did not increase the expressions of these mitochondrial marker proteins in the soleus muscle (data not shown).

### 2.5. The Exercise-Induced Increase in the Expression of Mitochondrial Biogenesis Inducers in Skeletal Muscle Was Reduced in TLR4m Mice

**Figure 4:**
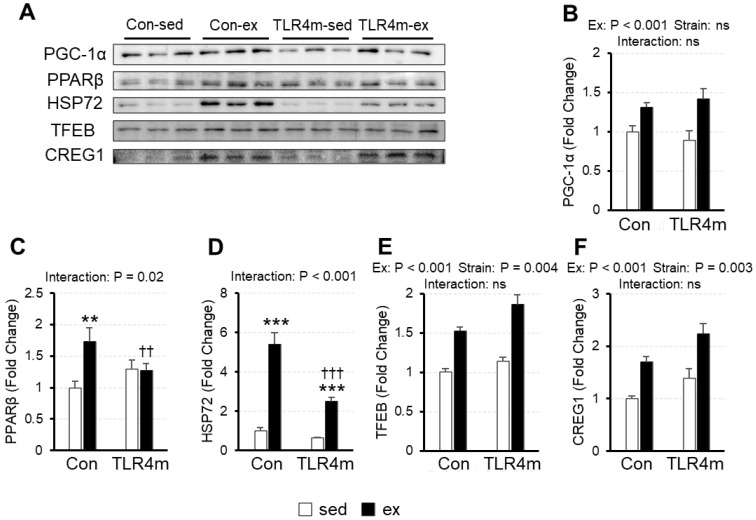
The relative expressions of molecules related to mitochondrial biogenesis in the plantaris. Peroxisome proliferator-activated receptor gamma co-activator 1-alpha (PGC-1α) (**B**), peroxisome proliferator-activated receptor beta (PPARβ) (**C**), heat shock protein 72 (HSP72) (**D**), transcription factor EB (TFEB) (**E**), and cellular repressor of E1A-stimulated genes 1 (CREG1) (**F**) were measured. Representative images of immunoblots are shown (**A**). Values are means ± standard error; *n* = 6 per group. *** *p* < 0.001 and ** *p* < 0.01 vs. sed within each strain; ††† *p* < 0.001 and †† *p* < 0.01 vs. controls within each condition.

To determine the molecular adaptations related to mitochondria, we measured the expression of mitochondrial biogenesis inducers, including peroxisome proliferator-activated receptor gamma co-activator 1-alpha (PGC-1α), peroxisome proliferator-activated receptor beta (PPARβ), heat shock protein 72 (HSP72), transcription factor EB (TFEB), and cellular repressor of E1A-stimulated genes 1 (CREG1) (Figure 4) in the plantaris. There was a significant interaction between exercise and strain in PPARβ (*p* = 0.02) (Figure 4C) and HSP72 (*p* < 0.001) (Figure 4D); exercise increased the expression of these proteins in the controls, whereas the increased effects were attenuated in the TLR4m mice. A significant main effect of exercise but not of strain was observed for PGC-1α (*p* < 0.001) (Figure 4B); exercise increased PGC-1α expression. The main effects of exercise and strain were observed for TFEB (*p* < 0.001, *p* = 0.004, respectively) (Figure 4E) and CREG1 (*p* < 0.001, *p* = 0.003, respectively) (Figure 4F); exercise increased the expression of these inducers, and the expression was higher in the TLR4m mice.

### 2.6. An Exercise-Induced Increase in Glucose Transporter 4 and Antioxidant Proteins in Skeletal Muscle Was Observed in the Controls and TLR4m Mice

**Figure 5:**
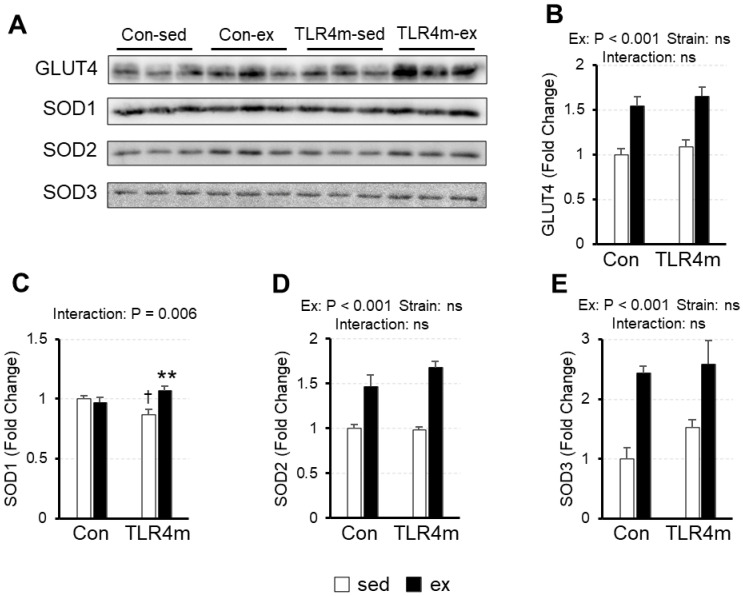
The relative expressions of molecules related to glucose metabolism and oxidative capacity in the plantaris. Glucose transporter 4 (GLUT4) (**B**), superoxide dismutase 1 (SOD1) (**C**), SOD2 (**D**), and SOD3 (**E**) were measured. Representative images of immunoblots are shown (**A**). Values are means ± standard error; *n* = 6 per group. ** *p* < 0.01 vs. sed within each strain; † *p* < 0.05 vs. controls within each condition.

To determine the molecular adaptations related to glucose metabolism and antioxidant capacity, we measured the expression of glucose transporter 4 (GLUT4), superoxide dismutase (SOD) 1, SOD2, and SOD3 (Figure 5) in the plantaris. There were significant main effects of exercise only in GLUT4 (*p* < 0.001) (Figure 5B), SOD2 (*p* < 0.001) (Figure 5D), and SOD3 (*p* < 0.001) (Figure 5E); exercise increased the expression. The increase in SOD1 following exercise was observed only in the TLR4m mice (*p* = 0.006) (Figure 5C).

### 2.7. The Transition of Fast to Slow-Type Myosin Heavy Chain Isoforms in Skeletal Muscle Was Induced by Exercise in the Controls and TLR4m Mice

**Figure 6:**
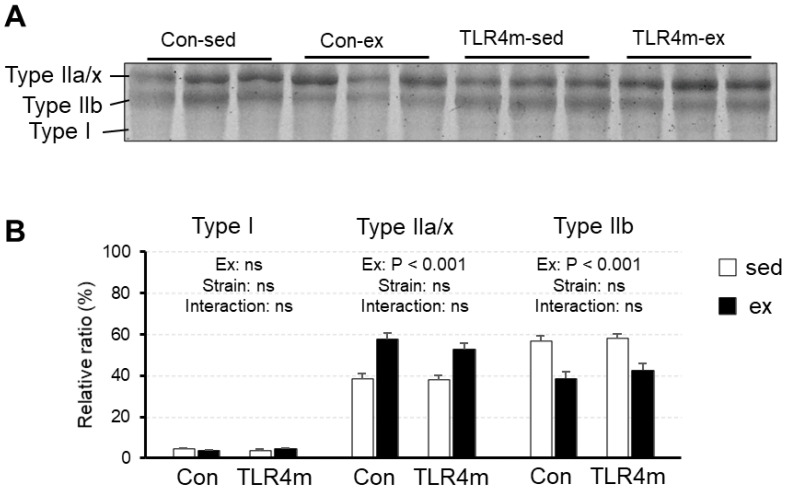
The relative expressions of myosin heavy chain (MHC) isoforms (**B**) in the plantaris. Representative images are shown (**A**). Values are means ± standard error; *n* = 6 per group. Differences between the groups were considered to be significant if *p* < 0.05.

To examine the exercise-induced muscle fiber-type adaptation, we measured the expression of myosin heavy chain (MHC) isoforms in the plantaris (Figure 6). There was a significant main effect of exercise on MHC type IIa/x (*p* < 0.001) and IIb (*p* < 0.001) but not of strain (*p* = 0.33 and *p* = 0.38, respectively); exercise training increased the proportion of MHC type IIa/x and decreased that of type IIb. No significant changes were observed in MHC type I.

## 3. Discussion

The results of the present study revealed several novel findings regarding the effect of the inflammatory response on exercise adaptation in mouse skeletal muscle. First, exercise upregulated the expression of various cytokines in skeletal muscle in the controls, but little increase was observed in the TLR4m mice (Figure 2). Second, exercise increased the expression of the mitochondrial markers and biogenesis inducers PPARβ and HSP72 in the controls, whereas the increased effects were reduced in the TLR4m mice (Figure 3 and Figure 4C,D). Third, the exercise-induced increase in the expression of PGC-1α, TFEB, CREG1, GLUT4, and antioxidant proteins and the ratio of slow-type MHC were identical between the controls and the TLR4m mice (Figure 4B,E,F, Figure 5, and Figure 6). 

Previous studies showed that cytokines increase after acute exercise [9,10]. In this study, six-week voluntary wheel-running increased the expression of various cytokines in the controls (Figure 2), which suggests that endurance exercise induces inflammation in skeletal muscle. One of the receptors which operates inflammation, TLR4, combined with its ligands activates NF-κB through myeloid differentiation factor 88 (MyD88) and IL-1 receptor-associated kinase-4 (IRAK-4), and thereby produces cytokines such as TNF-α, IL-1β, and IL-6 [16,22]. In the present study, exercise scarcely increased cytokines in TLR4m. It is possible that processes regarding MyD88, IRAK-4, and NF-κB are altered in TLR4m mice, which attenuated the exercise-induced increase of cytokine expressions.

Mitochondria are organelles that produce ATP through oxidative phosphorylation. The supply of ATP to muscles is essential for exercise, and exercise is a powerful stimulus that increases the expression of mitochondrial proteins and their oxidative capacity [5,8]. However, the molecular mechanism of exercise-induced mitochondrial biogenesis is not fully understood. In this study, the expression of mitochondrial marker proteins was increased in exercised mice, whereas the adaptations were attenuated in the TLR4m mice except for SDHB (Figure 3F) and NDUFB8 (interaction; *p* = 0.05) (Figure 3E), which suggests that exercise-induced mitochondrial biogenesis is mediated via TLR4. Alternatively, mitochondrial markers did not increase in the soleus muscle of exercised mice. Previous studies also showed that two or four weeks of voluntary wheel running did not increase mitochondrial proteins in the soleus muscle [25,26,27].

PGC-1α is a well-known key regulator of several aspects of energy metabolism; however, the role of PGC-1α in exercise-induced mitochondrial adaptations is still controversial. Several studies have reported that PGC-1α regulates exercise-induced mitochondrial biogenesis in skeletal muscle [28,29], while others have reported that PGC-1α is unnecessary because exercise-induced mitochondrial biogenesis in skeletal muscle was not diminished in muscle-specific PGC-1α knockout mice [27,30]. In this study, the exercise-induced adaptation of mitochondrial proteins was attenuated in the TLR4m mice despite the fact that the adaptation of PGC-1α was identical between the controls and TLR4m mice (Figure 3 and Figure 4B), which suggests that the exercise-induced increase in PGC-1α is not regulated by TLR4, and that proteins other than PGC-1α are involved in exercise-induced mitochondrial biogenesis through TLR4.

PPARβ (PPARβ/δ) is a nuclear receptor transcription factor and has multifaceted roles in development, lipid metabolism, energy expenditure, tissue repair and regeneration, and inflammation [31]. In recent years, PPARβ has emerged as a key transcription factor in skeletal muscle biology. Transgenic mice with muscle-specific overexpression of PPARβ had increased expression of mitochondrial enzymes without an increase in PGC-1α mRNA [32]. It has also been suggested that mitochondrial markers and mitochondrial enzymes were increased 10 days after PPARβ overexpression in skeletal muscle, whereas PGC-1α was not [33]. In this study, PPARβ expression following exercise was increased in the skeletal muscle of the controls, whereas the increased adaptation was attenuated in the TLR4m mice (Figure 4C), in accordance with mitochondrial marker adaptation (Figure 3). Therefore, PPARβ is a possible factor that regulates TLR4-mediated exercise adaptations.

HSPs have several isoforms and protect cellular homeostasis from stresses such as thermal stress and ultraviolet exposure. HSP72 is the inducible form of the 70-kDa family of HSPs, which is reported to be induced by exercise [34,35]. In this study, HSP72 expression was increased in the exercised mice, and the adaptation was attenuated in the TLR4m mice (Figure 4C), which suggests that the exercise-induced increase in HSP72 is regulated by TLR4. HSP72 has also been reported to increase the number of mitochondria and oxidative capacity in rodent skeletal muscle [36]. In this study, the increase in HSP72 following exercise was suppressed in the TLR4m mice (Figure 4D) in accordance with mitochondrial markers (Figure 3). Therefore, HSP72 is likely involved in TLR4-mediated exercise adaptation.

TFEB is a transcription factor that regulates autophagy [37,38]. Recent studies revealed that TFEB, along with PGC-1α, is an important regulator of exercise-induced mitochondrial biogenesis in skeletal muscle [39]. Another factor, the secreted glycoprotein CREG1, has recently been suggested to modulate mitophagy and improve exercise performance [40]. In this study, exercise increased the expression of TFEB and CREG1 in the controls and TLR4m mice (Figure 4E,F), which suggests that TFEB and CREG1 are not regulators for TLR4-mediated exercise adaptations. This is the first report to show the expression change of CREG1 in response to exercise.

There are several limitations in the present study. First, we examined expression of mitochondrial marker proteins but did not provide physiological data on the mitochondria such as citrate enzyme activity. Second, it would be better to discuss the exercise data such as running distance and speed. In previous research, there was no difference in the voluntary wheel-running exercise data between the control mice and TLR4m mice [41,42]. Finally, it is also required to clarify how exercise-induced adaptations were attenuated in the TLR4m mice. Although PPARβand HSP72 were identified as being involved in TLR4-mediated exercise adaptation, the mechanism has not been clear. To investigate them, further research is anticipated.

In summary, a voluntary wheel-running exercise over six weeks increased the expression of inflammatory cytokines, mitochondrial marker proteins, and mitochondrial biogenesis inducers such as PPARβ and HSP72 in the skeletal muscle of TLR4-active mice. However, the adaptations were attenuated in TLR4-inactive mice. Our findings suggest that exercise adaptations, especially mitochondrial biogenesis are mediated via TLR4. Additionally, TLR4-mediated inflammatory responses could be involved in the adaptation mechanism.

## 4. Materials and Methods

### 4.1. Animals

Six-week-old male C3H/HeN (intact TLR4 signaling/control) and C3H/HeJ (TLR4 mutant/TLR4m) mice were purchased from Shimizu Breeding Laboratories (Kyoto, Japan). The mice were placed in a room maintained at 22–24 °C with a 12 h:12 h light/dark cycle and fed a standard diet (Certified Diet NMF; Oriental Yeast, Tokyo, Japan) and water ad libitum.

All animal protocols were performed in accordance with the Guide for the Care and Use of Laboratory Animals by the National Institutes of Health (Bethesda, MD, USA).

### 4.2. Lipopolysaccharide Injection

The two types of mice were each divided randomly into two groups (*n* = 2–3 per group; from the viewpoint of animal protection, the number of animals was kept to a minimum): LPS injection (LPS+) and non-injection controls (LPS−). The LPS+ group was injected intraperitoneally with LPS (FUJIFILM Wako Pure Chemical, Osaka, Japan) dissolved in saline without anesthesia at 0.4 mg/kg body weight. Three hours after the injection, the mice were euthanized by cervical dislocation. As a control, each type of non-injected mouse was also euthanized in the same manner. Immediately after the euthanization, the plantaris muscle was dissected and rapidly frozen and then stored at −80 °C until analysis.

### 4.3. Real-Time Polymerase Chain Reaction Analysis

The mRNA levels of genes were quantified using real-time polymerase chain reaction (PCR) analysis, which was performed as described previously [43]. Total RNA from the plantaris was extracted using the RNeasy Plus Mini Kit (Qiagen, Hilden, Germany) according to the manufacturer’s instructions. The cDNA was reverse transcribed from 300 ng of total RNA using the PrimeScript RT Master Mix (Takara Bio, Kusatsu, Japan). Synthesized cDNA was then subjected to real-time PCR using the Applied Biosystems StepOne (Applied Biosystems, Foster City, CA, USA) with TB Green Premix Ex Taq II (Takara Bio). All PCR cycles was performed as follows: initial denaturation at 95 °C for 30 s followed by 40 cycles of 95 °C for 5 s; 60 °C for 30 s with specific primers. The mRNA levels of TNF-α, IL-1β, and IL-6 were normalized to the mRNA levels of glyceraldehyde 3-phosphate dehydrogenase (GAPDH) as an endogenous housekeeping control. The following primer sets were used:

GAPDH, 5′-TGTGTCCGTCGTGGATCTGA-3′ (forward),
GAPDH, 5′-TTGCTGTTGAAGTCGCAGGAG-3′ (reverse),
TNF-α, 5′-ACTCCAGGCGGTGCCTATGT-3′ (forward),
TNF-α, 5′-GTGAGGGTCTGGGCCATAGAA-3′ (reverse),
IL-1β, 5′-TCCAGGATGAGGACATGAGCAC-3′ (forward),
IL-1β, 5′-GAACGTCACACACCAGCAGGTTA-3′ (reverse),
IL-6, 5′-CCACTTCACAAGTCGGAGGCTTA-3′ (forward),
IL-6, 5′-TGCAAGTGCATCATCGTTGTTC-3′ (reverse)

### 4.4. Voluntary Wheel-Running Exercise

The voluntary wheel-running exercise procedure followed the previous study [26]. After one week of acclimatization, the two types of mice were each divided randomly into two groups (*n* = 6 per group): sedentary (sed) and exercise (ex). The four groups in total were housed in plastic cages (*n* = 3 per cage) with (ex) or without (sed) a plastic running wheel for six weeks. Body weight was measured each week. At the end of the experiment, the mice were euthanized. Immediately after that, the plantaris, soleus, and epididymal fat were removed, frozen in liquid nitrogen, and stored at −80 °C until analysis.

### 4.5. Western Blot Analysis

A western blot analysis was performed as described previously [43], with modifications. Frozen plantares were homogenized in ice-cold buffer (1:40, wt/vol) containing 20 mmol/L Tris-HCL (pH 7.4), 1% Triton X-100, 50 mmol/L sodium chloride, 250 mmol/L sucrose, 50 mmol/L sodium fluoride, 5 mmol/L sodium pyrophosphate, 2 mmol/L dithiothreitol, 50 mg/L soybean trypsin inhibitor, 4 mg/L leupeptin, 1 mmol/L sodium orthovanadate, 0.5 mmol/L phenyl-methyl-sulfonyl-fluoride, and 0.1 mmol/L benzamidine and centrifuged at 16,000× *g* for 40 min at 4 °C. The supernatants were collected and the protein content determined using the Bradford technique (Protein Assay CBB Solution, Nacalai Tesque, Kyoto, Japan). The supernatants were then solubilized in a Laemmli sample buffer. The samples (10 µg of protein) with or without boiling for 5 min were separated by sodium dodecyl sulfate (SDS)-polyacrylamide gel and transferred to polyvinylidene difluoride membranes (Millipore, Billerica, MA, USA). The membranes were blocked with nonfat dry milk (Nacalai Tesque) and incubated with primary antibodies: GLUT4 (#4670-1704; Bio-Rad Laboratories, Hercules, CA, USA), PGC-1α (#ab54481; Abcam, Cambridge, UK), HSP72 (#ADI-SPA-812; Enzo Life Science, NY, USA), OXPHOS (#ab110413; Abcam), PPARβ (#sc-74517; Santa Cruz Biotechnology, Dallas, TX, USA), TFEB (#13372-1-AP; Proteintech, BWChicago, IL, USA), CREG1 (#12220-1-AP; Proteintech), SOD 1 (#AF3787-SP; R&D Systems, Minneapolis, MN, USA), SOD2 (#AF3419-SP; R&D Systems), and SOD3 (#AF4817-SP; R&D Systems). The membranes were washed with Tris-buffered saline-Tween (TBS-T) and reacted with secondary antibodies: anti-rabbit IgG (Cell Signaling, Technology, Danvers, MA, USA), anti-mouse IgG (Cell Signaling Technology), or anti-goat IgG (Jackson ImmunoResearch Laboratories, West Grove, PA, USA) for 1 h at room temperature. After a final wash with TBS-T, the protein bands were visualized using Chemi-Lumi One (Nacalai Tesque). The signal density was measured using a WSE-6100 LuminoGraph (ATTO, Tokyo, Japan). Each sample was investigated in duplicate, at least, and each averaged density was used as a datum to ensure that results were not influenced by loading errors. The mean intensity of basal samples in each membrane was used as a reference for controlling gel-to-gel variation.

### 4.6. Cytokine Array

The changes in proinflammatory cytokines among the four groups were quantified using the Mouse Cytokine Array Panel A (R&D Systems), according to the manufacturer’s instructions. In this experiment, two plantaris samples from a same group were mixed into one sample; thus, there were three samples for each group. Data were normalized to the reference spots of the same membrane. The ratio of control-ex to control-sed and TLR4m-ex to TLR4m-sed were then calculated to compare them.

### 4.7. Measurement of Myosin Heavy Chain Isoform Composition

The MHC isoform composition (I, IIa/IIx, and IIb) was analyzed using a modification of previously described methods [43]. In brief, the boiled samples (5 µg of protein) were separated on SDS-polyacrylamide (7%) gel at 120 V for 19 h in a temperature-controlled chamber at 4 °C. After electrophoresis, the gels were stained with Oriole™ Fluorescent Gel Stain (Bio-Rad Laboratories). The gels were visualized using ImageCapture G3 (Liponics, Tokyo, Japan) and analyzed using ImageJ software (National Institutes of Health, MD, USA).

### 4.8. Statistical Analysis

The data are expressed as means ± standard error. The two mean values for the controls and TLR4m mice were compared with a Student’s t-test in the LPS injection experiment. In the other experiments, multiple mean values were compared using a two-way analysis of variance with strain (controls vs. TLR4m) and exercise (sed vs. ex) as the main factors, followed by the Tukey-Kramer post hoc test. Differences between the groups were considered significant for *p*-values < 0.05.

## Figures and Tables

**Table 1:** Body weight, plantaris muscle weight, and epididymal fat weight.

	Controls		TLR4m		
	sed	ex	sed	ex	
BW (initial)	22.66 ± 0.45	23.13 ± 0.43	22.04 ± 0.48	22.11 ± 0.34	
BW (final)	27.53 ± 1.06	26.9 ± 0.42	24.88 ± 0,88	23.5 ± 0.36	strain: *p* < 0.001
PLA/BW	0.57 ± 0.03	0.60 ± 0.03	0.56 ± 0.01	0.64 ± 0.01	ex: *p* = 0.006
EFAT/BW	14.68 ± 1.79	3.07 ± 0.78	14.61 ± 2.02	6.43 ± 0.79	ex: *p* < 0.001

The initial and the final body weight (BW), plantaris muscle (PLA) weight normalized to body weight (BW), and epididymal fat (EFAT) weight normalized to the BW of mice in each group. Two strains of mice, TLR4 mutant (TLR4m) and controls, were divided into a voluntary wheel-running group (ex) and a sedentary group (sed), respectively, and housed for six weeks. Values are means ± standard error; *n* = 6 per group. Differences between the groups were considered to be significant if *p* < 0.05.

## Data Availability

The data that support the findings of this study are available from the corresponding author, upon reasonable request.
